# Revisiting Wetting, Freezing, and Evaporation Mechanisms of Water on Copper

**DOI:** 10.1021/acsami.1c09733

**Published:** 2021-07-28

**Authors:** Emil Korczeniewski, Paweł Bryk, Stanisław Koter, Piotr Kowalczyk, Wojciech Kujawski, Joanna Kujawa, Artur P. Terzyk

**Affiliations:** † Faculty of Chemistry, Physicochemistry of Carbon Materials Research Group, Nicolaus Copernicus University in Toruń, Gagarina Street 7, 87-100 Toruń, Poland; ‡ Faculty of Chemistry, Chair of Theoretical Chemistry, Maria Curie - Skłodowska University, 20−031 Lublin, Poland; § Faculty of Chemistry, Department of Physical Chemistry and Physical Chemistry of Polymers, Nicolaus Copernicus University in Toruń, Gagarina Street 7, 87-100 Toruń, Poland; ∥ College of Science, Health, Engineering and Education, Murdoch University, Perth, Western Australia 6150, Australia

**Keywords:** contact angle, wetting, copper, freezing, evaporation, molecular dynamics

## Abstract

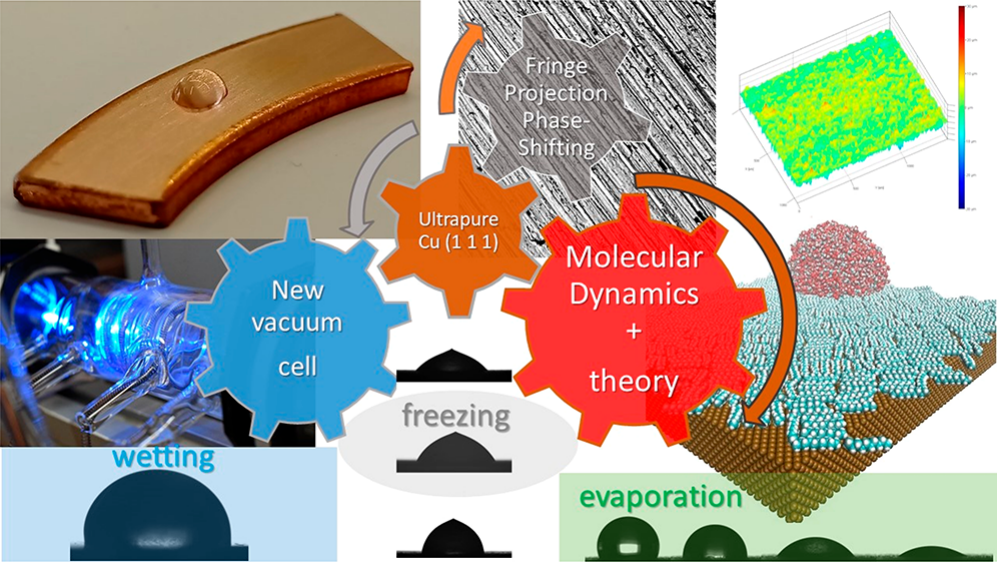

Wetting of metal surfaces plays an important role in fuel cells, corrosion science, and heat-transfer devices. It has been recently stipulated that Cu surface is hydrophobic. In order to address this issue we use high purity (1 1 1) Cu prepared without oxygen, and resistant to oxidation. Using the modern Fringe Projection Phase-Shifting method of surface roughness determination, together with a new cell allowing the vacuum and thermal desorption of samples, we define the relation between the copper surface roughness and water contact angle (WCA). Next by a simple extrapolation, we determine the WCA for the perfectly smooth copper surface (WCA = 34°). Additionally, the kinetics of airborne hydrocarbons adsorption on copper was measured. It is shown for the first time that the presence of surface hydrocarbons strongly affects not only WCA, but also water droplet evaporation and the temperature of water droplet freezing. The different behavior and features of the surfaces were observed once the atmosphere of the experiment was changed from argon to air. The evaporation results are well described by the theoretical framework proposed by Semenov, and the freezing process by the dynamic growth angle model.

## 1 Introduction

Surface hydrophilicity/hydrophobicity plays an important role in everyday life and in many industrial applications, including the clothing, building, and aviation industries, where superhydrophobic, superomniphobic, self-cleaning, and anti-icing surfaces^1^ are widely applied. Metal surface wetting is also a very important aspect in fuel cells,^2^ in corrosion science,^3^ or even in heat-transfer devices.^4^ On the other hand, the determination of hydrophilicity/hydrophobicity is not a simple task because it is strongly affected by surface contaminations.^5,6^ For example, airborne hydrocarbons adsorbed on a surface strongly influence the water contact angle (WCA) on Au,^7^ PTFE,^6^ Si,^8^ graphene,^5,9^ and many other surfaces. It was also proven that the mechanism of wetting is strongly related, among other things, to the affinity between a substrate surface to a droplet and to adsorbed hydrocarbons. The residual contact angle value is determined by a simple balance between the work of droplet adhesion to a bare substrate surface and the work of adhesion to a surface covered by adsorbed hydrocarbons.^6^

Among the studies reported in the literature, there are discrepancies in WCA values on copper. For example, it was suggested that the most probable WCA for a smooth copper surface is 52°,^10^ although some authors reported smaller values.^4,11^ Recently it has been also postulated that a copper surface is strongly hydrophobic.^12^ Therefore, one can expect, similarly like in the case of PTFE,^13^ that a simple roughening of a copper surface should lead to the rise in WCA according to the Cassie–Baxter mechanism. In fact, the static WCA for the smooth copper surface was recently reported as close to 99° and, as expected, strongly increased for a surface roughened by using different methods.^12^ However, the majority of wetting studies do not consider the presence of airborne hydrocarbons, the authors do not provide the substrate characterization (for example, the roughness factor is not provided, and this has a crucial influence on WCA value), and do not use sufficient surface cleaning.

To settle the issue of whether the WCA on pure copper surface is smaller or larger than 90° one can resort to the literature data reporting water adsorption studies on copper since it is well proven that the shape of water adsorption isotherm is strongly related to the surface hydrophobicity.^14^ Following the BET/IUPAC adsorption isotherms classification,^15^ if water adsorption isotherm is of the type III and/or V one can expect a small affinity of water to a surface, as it has been observed (for example) for strongly hydrophobic unmodified carbonaceous materials.^14^ The appearance of polar sites, for instance oxygen surface functionalities, on a hydrophobic surface leads to the rise of water-surface affinity at low and intermediate relative pressures. As a result, water adsorption isotherm changes its shape, and type II or IV is observed.^14^ Considering the results reported for copper by Lee and Staehle,^16^ and/or Sharma and Thomas,^17^ one can conclude that water adsorption isotherms on copper are of the II type, thus a pure Cu surface is hydrophilic.

One of the aims of this study was to investigate what is the WCA on a well-characterized (1 1 1) copper surface, possessing strictly defined roughness. To do so, an ultrahigh purity copper was used, and a special cell was constructed, enabling the contact angle measurements in hydrocarbons-free atmosphere and after the long-term vacuum and thermal cleaning of a surface. Applied cleaning procedure eliminates hydrophobic and hydrophilic centers from a copper surface. Using the modern Fringe Projection Phase-Shifting method of surface roughness determination, we define the relation between copper surface roughness and WCA, and next, by a simple extrapolation, we determine the WCA for the perfectly smooth copper surface. Additionally, the kinetics of airborne hydrocarbons adsorption on copper was measured. It is shown for the first time that the presence of surface hydrocarbons has a strong impact not only on the WCA values but also on water droplet evaporation and the temperature of water droplet freezing. To get an insight into the influence of airborne hydrocarbons we support the experimental results by molecular dynamics simulation data, and modern theoretical approaches.

## 2 Materials and Methods

### 2.1 Copper Samples Preparation and Characterization

As a source of the initial copper, a vacuum gasket made of OFHC (oxygen free high conducted) copper (Artvac, Poland) was applied. This gasket, dedicated to ultrahigh vacuum (UHV) systems, contains the highest purity grade of copper (99.99%) prepared without oxygen access and possessing very high electric conductivity. What is also important, this copper contains (1 1 1) planes. This is crucial for wetting studies because it is well proven that (1 1 1) copper is resistant to oxidation (in fact, the oxidation of this plane is negligible) and prevents water dissociation. This is crucial for wetting studies because it is well proven that (1 1 1) copper is, among all Cu surfaces the least reactive in reaction with oxygen, and hence the most resistant to oxidation (in fact, the oxidation of this plane is negligible). Cu (111) surfaces also prevents water dissociation.^18^ This structure is confirmed by XRD spectra shown in Supporting Information (SI) Figure S1. The spectra were obtained using Philips X’Pert in a transmission mode over a 5–120° 2θ range with the scanning speed equal to 0.05 deg/min using X’Celerator Scientific detector with Cu anode (Malvern Panalytical, Malvern, UK). The diffraction profiles were corrected by linear plotting of background and then by the smoothing cycles. The contribution of Kα2 was eliminated by the Raschinger method. The established experimental data are in good accordance with the pattern (00–0040–0836). The appeared peaks 43.6°, 50.5°, 74.4°, 90.0°, 95.3°, and 116.8° are coming from the following reflections 111, 200, 220, 311, 222, and 400, respectively (see SI Figure S1). However, the peak at 43.6° shows the 111 reflection and possesses the I equal to 100%.

The initial sample was labeled as *Cu-0*. From this sample, two additional copper samples were obtained using a sandpaper (Trading - Stolker, Poland) of grit 600 and 2000, and the samples were labeled as *Cu-600* and *Cu-2000*, respectively (to remove the sandpaper residues and Cu dust the samples were cleaned using Ar stream–for the efficiency of this procedure see SI Figure S2).

For data acquisition and analysis, One Attension software was utilized equipped with Attension 3D Topography Module (Biolin Scientific Oy, Espoo, Finland). The advantage of the method is the possibility to determine the apparent and intrinsic contact angle (corrected by roughness). To assess the wettability of the surfaces, the dynamic contact angle measurements were performed. The water droplet (6 μL) was placed by dropping it from the tip of the needle from the height of 1.5 mm and its behavior was monitored during no more than 1000 s. To distinguish which process occurred, that is, evaporation and/or water wetting, the evolution of following factors were determined: WCA, volume and height of the testing liquid, droplet base and work of adhesion.

### 2.2 Contact angle in Ar and in Air Atmosphere, Droplet Evaporation, and Freezing

**Figure 1:**
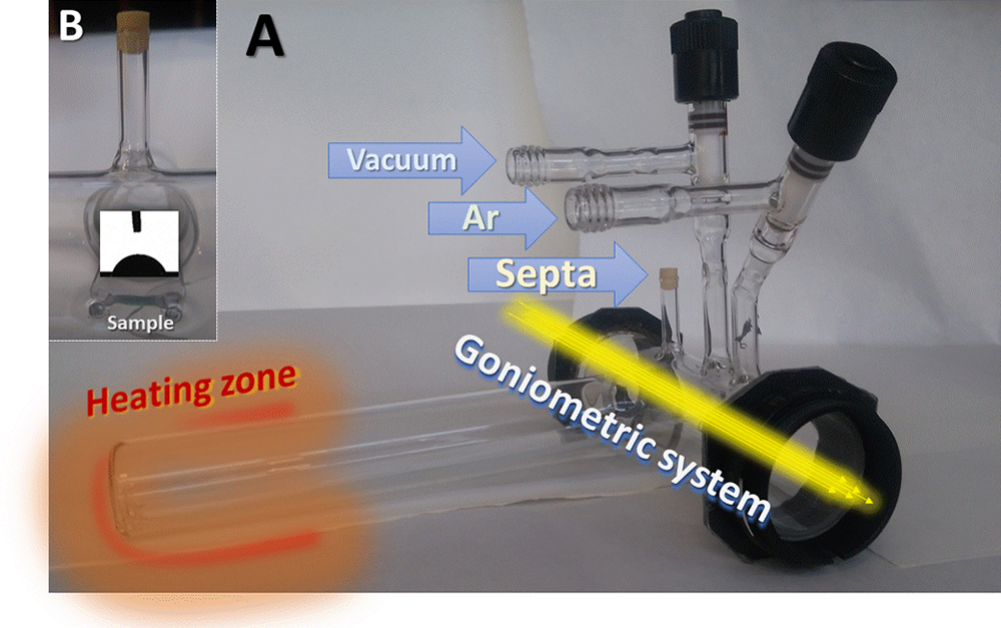
An overview of a new homemade cell for the measurement of contact angle in controlled atmosphere (A). The vacuum and Ar valves are shown together with the septa-supported dosing system. The goniometric system (a bright diode and lens) is placed on the optical axis crossing the both sides of two sight glasses. The inset (B) shows the side view and a typical image of WCA on a thermally desorbed under vacuum sample.

Before WCA measurement in Ar atmosphere each sample was placed separately in the special homemade cell (Figure 1).

Both sides of this cell are equipped with sight glasses enabling observation of three phase contact line using a goniometric system described in details previously.^8^ In short: it contains a camera Grasshopher3 GS3-U3–32S4C–C, 3.2 Mpx connected to the specially designed optical system consisting of perfectly located in the optical axis two elements: Edmund Optics 1.0x Telecentric Lens 55350 and Edmund Optics Telecentric Illuminator Lens 62760 with a bright diode placed in it (MicroBriteSpot/Coaxial Light model: SL223 470 IC, Light wavelength: 470 nm) leading to polarized parallel light. The cell was sealed up using FKM/FPM (Viton) gaskets and connected to two liquid nitrogen cold-trap reinforced vacuum pumps: Edwards E2M0.7 and a turbomolecular pump Leybold Turbovac 50. After placing a sample inside, it was desorbed for 24 h without heating to the pressure smaller than 1.2 × 10^–3^ mbar. After this preliminary desorption, a sample was further desorbed for subsequent 72 h at 673 K, and finally cooled down in a vacuum for 30 min in Ar atmosphere (Argon Premier 99.9992%, Air Products, Poland) at Ar pressure equal to 1 atm. Ar application prevents the introduction of oxygen into the system. The initial sample evacuation without heating is a very important step because in this way we avoid copper surface oxidation. Moreover, long-term heating supported by a sample evacuation eliminates not only hydrophobic but also hydrophilic contaminations, for example, polar gases present in air (CO_2_, adsorbed H_2_O etc.). The presence of hydrophilic centers can drastically decrease WCA^18^ even to the value equal to zero, as it was proven by White and Drobek.^19^ A stainless steel needle containing deionized and deoxidized water (ion conc. < 6 ppm) was introduced into the system by a silicone septa (Injection Rubber Plug 201-35584, Shimadzu, Japan) and WCA values were measured (at least for three droplets, each was placed by dropping it from the tip of the needle from the height of 1.5 mm) and the results were averaged.

Samples measured in this system are denoted by subscript *Vac*. Desorbed in the same way samples were removed from a cell and subjected to air atmosphere. Samples measured after that exposure are denoted by subscript *Vac-Air*. WCA values were determined to check the influence of adsorbed airborne hydrocarbons on copper wetting. In this case, we applied our goniometric system described above. To check the influence of hydrocarbons on droplet evaporation in air (25.0 ± 0.1 °C, relative humidity = 35%) we used Attension Theta Flex Optical Tensiometer (Biolin Scientific Oy, Espoo, Finland). A freshly prepared samples (*Cu-0* and *Cu-2000*) were used. In order to obtain the comprehensive characterization of the systems the droplets were also examined for the occurrence of frozen surfaces. The aim of this experiment was to check how the presence of adsorbed airborne hydrocarbons affects the icephobicity. The freezing experiments were performed in air atmosphere (at the pressure of 1 atm, the relative humidity of 50%). To do so a freshly prepared samples of *Cu-2000-Air* were used. Droplet freezing (the temperature profile is shown in SI Figure S3) were studied in air, at 25.0 ± 0.1 °C (relative humidity = 50%) using Attension Theta Optical Tensiometer (Biolin Scientific Oy, Espoo, Finland) equipped with DEBEN Coolstage (model AFM Nanosurf by Deben UK, Woolpit, Suffolk, United Kingdom). Droplet at these measurements were also placed by dropping it from the tip of the needle from the height of 1.5 mm.

### 2.3 Molecular Dynamics Simulation

The details of MD simulation procedure are given in the Supporting Information. Shortly: three Cu surfaces were modeled, namely (1 1 1), (0 0 1), and (1 1 0). The energy parameter ε_Cu–Cu_ was determined empirically by matching the experimental WCA for (1 1 1) surface. We use TIP4*P*/2005 water model.^20^ To estimate the influence of line tension we simulated spherical and cylindrical nanodroplets. In order to check the influence of airborne hydrocarbons, *n*-decane model was used with the interaction parameters taken from the OPLS-AA force field.^21^ The electrostatic interactions were evaluated using the Particle Mesh Ewald (PME) method. All simulations were carried out using OpenMM package^22−24^ with a Nose-Hoover thermostat (τ = 0.1) at the time step 0.002 ps. The rigidity of water was enforced using SHAKE algorithm.

## 3 Results and Discussion

### 3.1 Cu Surface—Hydrophobic or Hydrophilic?

**Figure 2:**
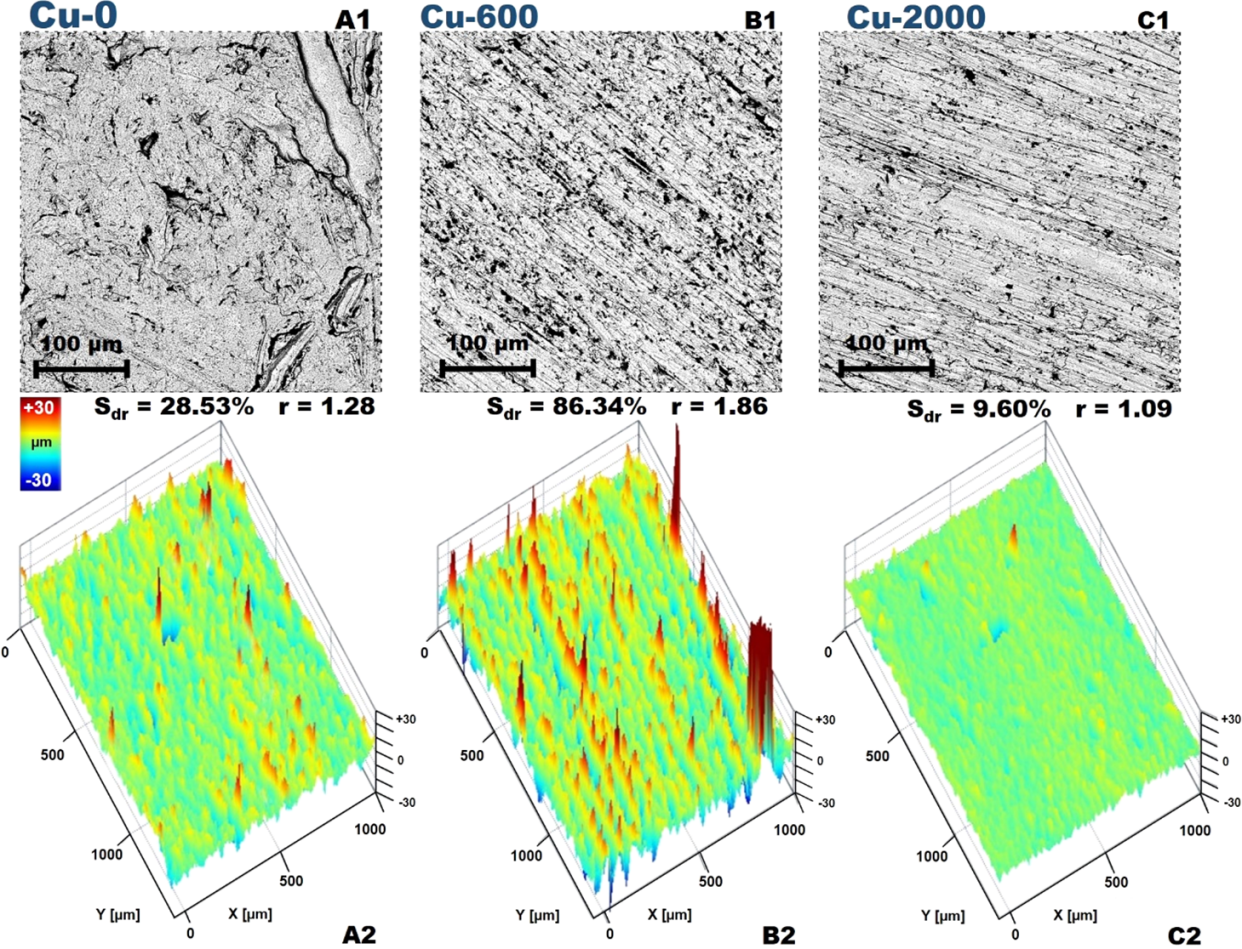
SEM images of *Cu-0* (A1), *Cu-600* (B1), *Cu-2000* (C1), and surface topography (A2–C2), together with respective roughness parameters *S*_DR_ and *r*.

In the materials science, to understand all changes caused by the modification, chemical, or physical, it is crucial to fully characterize the material. First the imaging of the surfaces and the roughness determination was performed. The pristine surface was characterized by homogeneous topography with little voids (Figure 2A1). After the treatment with sandpaper, significant changes have been noticed. Well-defined heterogeneities possessing the same orientations due to the roughening process were observed (Figure 2B1–C2). In the case of sandpaper usage with a grit size 600 (for *Cu-600* sample) much rougher material was generated. However, an interesting result was obtained for the copper treated with 2000 sandpaper (*Cu-2000* sample). The overall roughness of the latter sample was smaller than the pristine one. The roughness was expressed by the *S*_dr_ (area factor, the ratio between the interfacial and projected areas) (eq 1) and *r* (roughness ratio) (eq 2) which are roughness parameters according to the ISO 25178 standard. The roughness ratio is defined as the ratio between the actual and projected solid surface area (*r* = 1 for a smooth surface and *r* > 1 for a rough surface). The value of *r* equal to 1.09 for the *Cu-2000* sample proved that an almost totally smooth surface was generated.
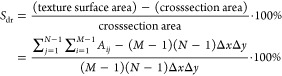
1
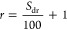
2where *S*_dr_ is surface area ratio, called also interfacial area ratio, (*M* – 1) (*N* – 1) Δ*x*Δ*y* – is the sampling area in the *XY* plane, ∑_*j* = 1_^*N*–1^ ∑_*j* = 1_^*M*–1^*A*_*ij*_ is the total area of the sample surface corresponding to the sampling area.

**Figure 3:**
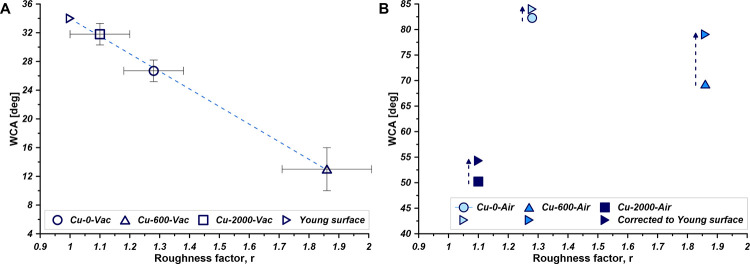
Relation between WCA determined in Ar (A) and air (B) atmosphere and the roughness factor. Young angle is determined for (1 1 1) Cu (extrapolation for *r* = 1) as equal to 34°. Corrected WCA values are also included (B).

In the next step of samples characterization, the roughness was correlated with WCA (Figure 3A). The linear relation between WCA and *r* proved the high quality of the roughening processes. The presented excellent correlation was observed for the materials desorbed and kept in the ambient atmosphere of Ar to ensure lack of surface oxidation and adsorbed airborne hydrocarbons. In the Ar atmosphere, the highly hydrophilic character of the copper surfaces was presented. WCA was ranging between 13.0° for *Cu-600-Vac* and 31.8° *Cu-2000-Vac*, respectively. The value of contact angle (WCA = 34°) for the totally smooth surface with *r* = 1, so-called Young surface, was calculated and added to the plot (Figure 3A). The WCA was determined from the Wenzel eq (eq 3):
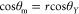
3where: *θ*_m_ is the measured contact angle, *θ*_*Y*_ is Young’s contact angle, and *r* is the roughness ratio.

When the atmosphere of the experiment was changed from argon to air different behavior and features of the surfaces were observed (Figure 3B). Importantly, the range of WCA switched from a highly hydrophilic to an almost hydrophobic one. Due to the lack of pure ambient atmosphere, the values of WCA were now placed between 50.2° and 82.3° (Figure 3B). Thanks to the implementation of Fringe Projection Phase- Shifting method (see SI Figures S4 and S5), it was possible to obtain the roughness–corrected WCA. The mentioned changes were dictated by the value of the roughness parameters. Namely, the biggest impact was noted for the *Cu-600* and the smallest one for *Cu-0* (Figure 3B).

**Figure 4:**
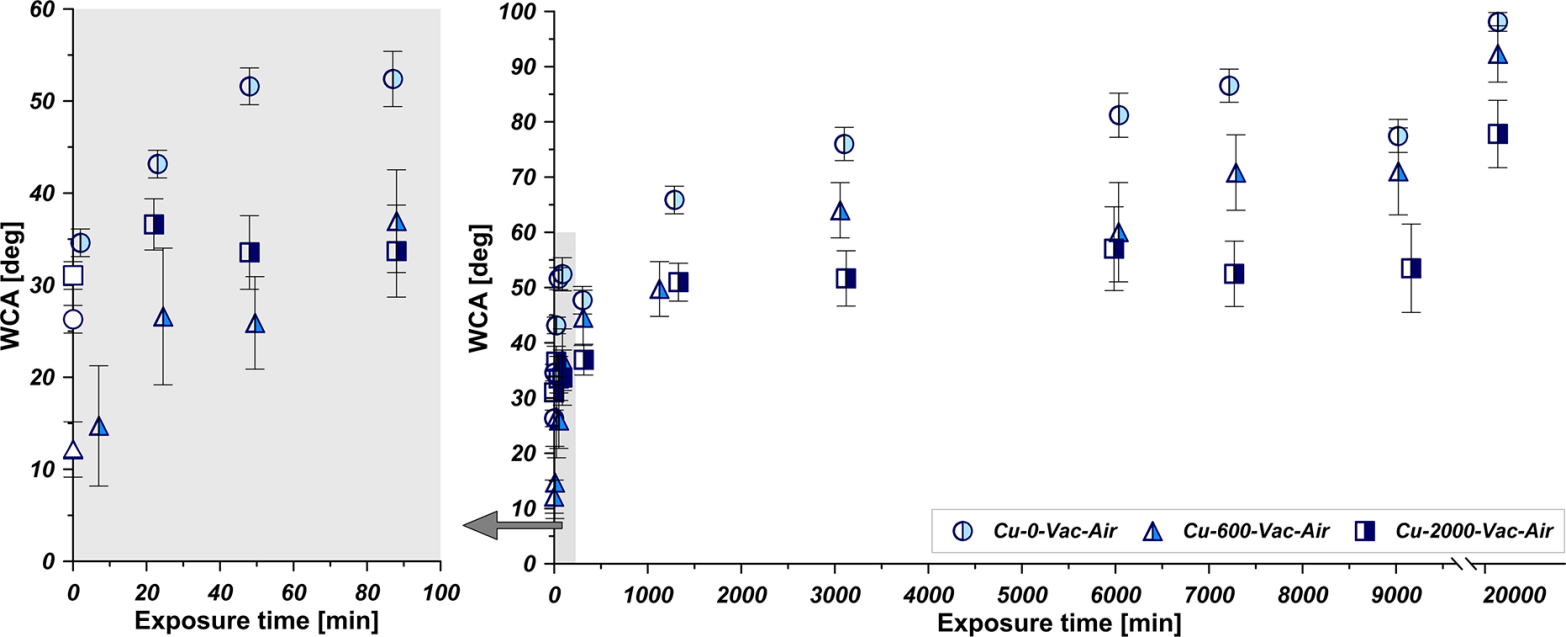
Influence of airborne hydrocarbons on WCA values for studied copper samples thermally desorbed under vacuum and subjected to air atmosphere.

Figure 4 collects the changes in the WCA of the samples thermally desorbed as in Figure 3A, but exposed directly to the atmosphere. It can be seen that the WCA increases with the exposure time changing the surface nature to less hydrophilic and even hydrophobic.

**Figure 5:**
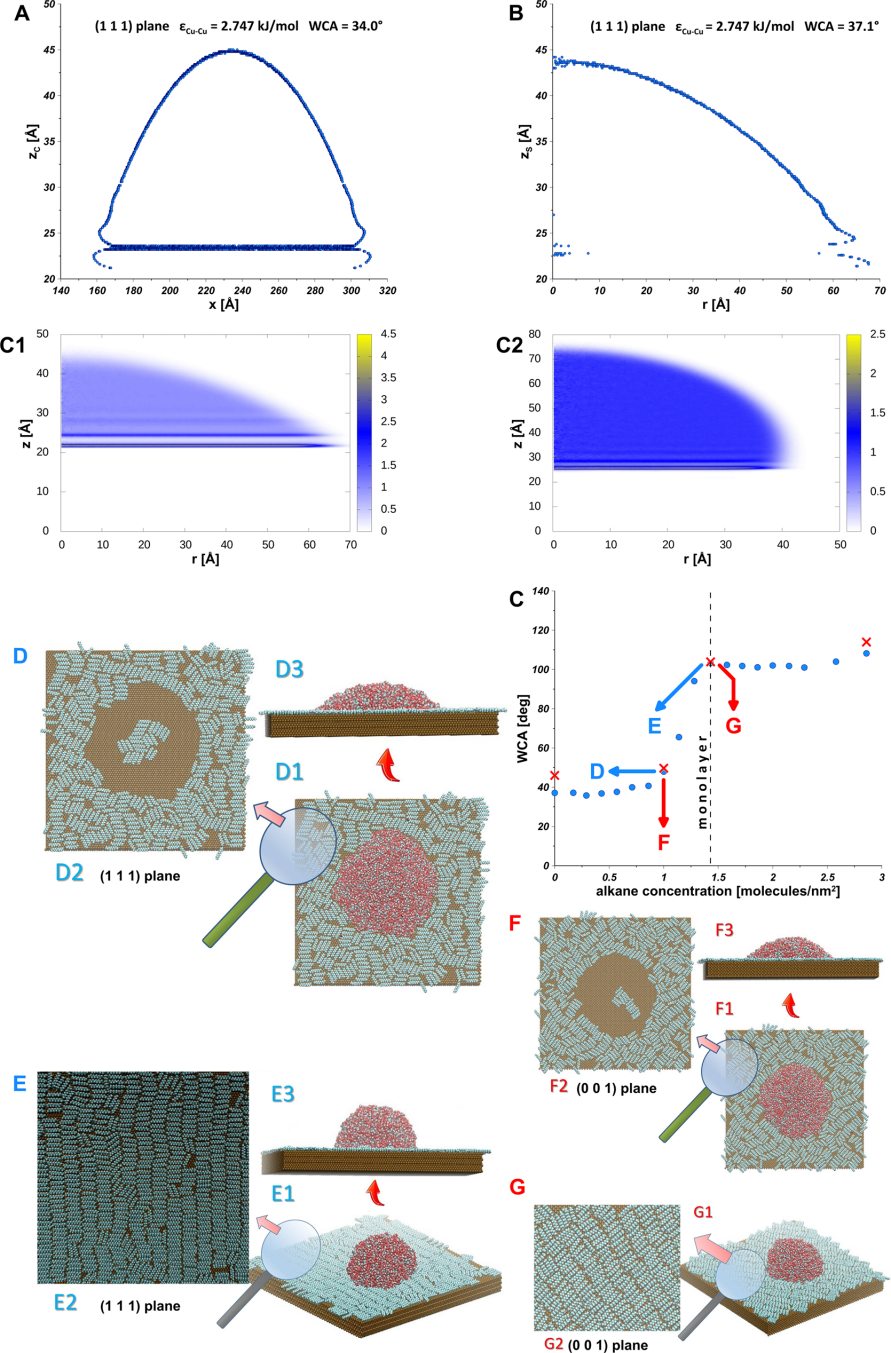
MD simulation results showing the wetting of (1 1 1) Cu surface-simulation of cylindrical (A) and spherical (B) droplet, the influence of adsorbed airborne hydrocarbons on WCA for Cu (1 1 1) (blue points) and Cu (0 0 1) (red crosses) surfaces (C), 2D densities of water on Cu (1 1 1) (C1) and Cu (1 1 1) covered with alkane monolayer (C2), and selected snapshots showing the droplet sitting on c.a. 0.7 (D1, D3) of monolayer and on monolayer (E1, E3) of hydrocarbons carpet on (1 1 1) surface. The snapshots for (0 0 1) are F1, F3, and G1 and G3, respectively. The orientation of hydrocarbons in 0.7 of monolayer and in monolayer is also shown for (1 1 1) (D2 and E2) and (0 0 1) Cu surfaces (F2 and G2). Snapshots were prepared using the VMD, a molecular visualization program.^25^

To get a deeper insight into the wetting mechanism we show in Figure 5A the results for 3900 TIP4*P*/2005 water molecules placed in a cylindrical droplet on the top of a perfect (1 1 1) Cu surface (after preemptive energy minimization the drop was equilibrated for 10 ns, and the averages were gathered for up to 40 ns). The solid–fluid interaction parameter was determined by matching the WCA of a cylindrical drop to the experimental value (34°, see Figure 3A), and the value equal to ε_Cu–Cu_ = 2.747 kJ/mol was found. The impact of droplet size on the WCA is very small (see the results presented in Figure 5B for the spherical droplet), thus we conclude about the small influence of the line tension on WCA. Figure 5C shows the simulation results of the effect of airborne hydrocarbons on WCA. It is interesting that WCA increases with the surface concentration of airborne hydrocarbons, reaching similar values as observed during the experiment (see Figure 4). We note that once the surface concentration of hydrocarbons gets over the monolayer concentration, the impact of additional *n*-decane molecules on WCA is quite small. Likewise, insignificant WCA changes can be observed for very small alkane concentrations. Figure 5D,F show the snapshots of water on *n*-decane covered (1 1 1) and (0 0 1) copper surface evaluated at surface concentration equal to 1 molecules/nm^2^, what corresponds to ca. 0.7 of hydrocarbon monolayer. The droplet is in so-called “dimple” state,^6^ that is, only few or no hydrocarbons under the droplet are observed. Figure 5E,G shows the snapshots of water on *n*-decane covered (1 1 1) and (0 0 1) copper surface evaluated at surface concentration equal to 1.45 molecules/nm^2^, what corresponds to a full hydrocarbon monolayer. We find the droplet to be in the so-called “carpet” state.^6^ It is worthy to mention, that we also performed simulations for (1 1 0) Cu surfaces. We assessed how different exposed planes affect the WCA values, and to what extent the change in the exposed surface type influences the packing of the adsorbed hydrocarbons (selected representative results are shown in Figure 5C for selected points of (0 0 1) surface). Our simulation data show that the WCA on pure (0 0 1) Cu surface for cylindrical droplet is equal to 44°, that is, this Cu plane is more hydrophobic than (1 1 1) plane. The influence of the airborne hydrocarbons is similar for both surfaces leading to similar WCA values as recorded for (1 1 1) surface (Figure 5C); however, small differences in WCA can be caused by small differences in hydrocarbons packing. Also the orientation of “carpet state” hydrocarbons on both Cu surfaces is the same (Figure 5E2,G2). Hydrocarbons energy minimization is achieved by parallel orientation on Cu surface.

**Figure 6:**
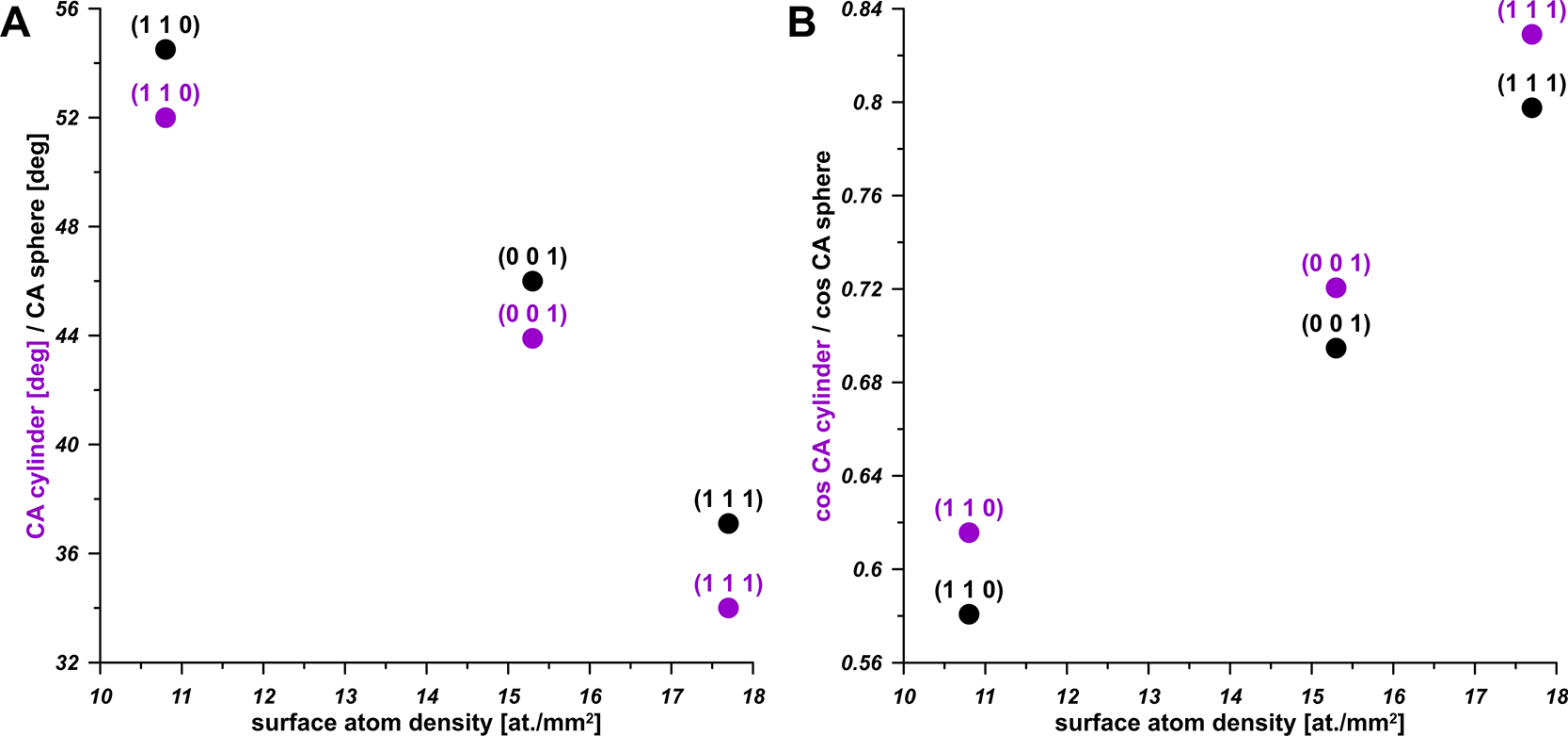
Correlations between Cu surface atom density and WCA (A), cos(WCA) (B) calculated for cylindrical and spherical droplets, based on MD simulation results.

Finally, Figure 6 collects the WCA values plotted as a function of the density of Cu surface atoms. Excellent correlations are recorded, leading to the conclusion that all Cu surfaces are hydrophilic, however, large differences in WCA are recorded. Thus, it can be observed, that predicted by us from MD simulation WCA values can vary for pure Cu in the range 34° (for (1 1 1)) to 52° (for (1 1 0)), with the value of 44° for Cu (0 0 1). This is correlated with the density of the surface atoms.

As the density of surface atoms increases, the rise in the work of droplet adhesion is observed, leading to the decrease in WCA value.

### 3.2 How Airborne Hydrocarbons Influence Droplet Evaporation on Cu?

**Figure 7:**
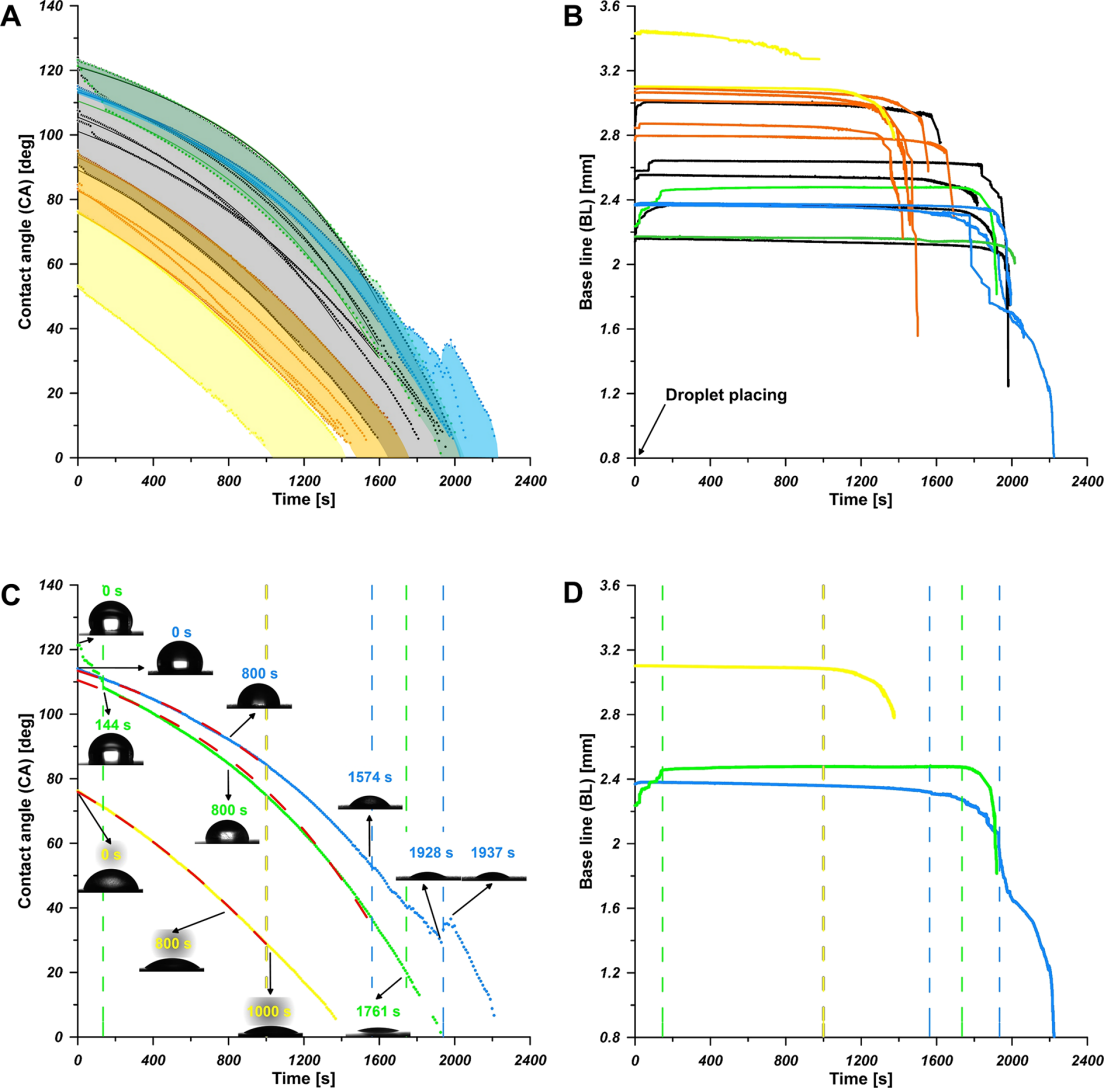
Change of WCA (A) and the evolution of droplet baseline (BL) during evaporation (B); dotted lines: experimental data, solid lines: fits according to eq 8 for selected curves, with the stages of evaporation (dashed lines with corresponding colors). The evolution of droplet BL during evaporation (D), (dashed lines as above). Color codes for all four figures: *Cu-2000-Air-0min* (yellow), *Cu-2000-Air-20min* (orange), *Cu-2000-Air-93h* (black), *Cu-2000-Air-8 mths* (bright green), *Cu-2000-Vac-Air-8 mths* (rotten green), *Cu-0-Air-infinity* (blue). The ranges of the same repeated measurements are marked with stripes in accordance with a color code on panel A.

Figure 7 collects the results of the experiment on droplet evaporation. Generally, one can observe that with the rise in exposure time, the WCA increases due to presence of hydrocarbons (Figure 7A). This presence, however, causes the elongation of the evaporation process (Figure 7A), accompanied by the decrease in baseline length (Figure 7B) and the work of adhesion (SI Figure S6).

According to the current state of art^26,27^ three stages of evaporation from the sessile water droplet in unsaturated H_2_O vapor atmosphere can be distinguished: the evaporation at a constant contact area and decreasing contact angle, at a decreasing contact area and constant contact angle, and finally, at both area and angle decreasing.

Because of the short time, the evaporation at the beginning (just after the droplet deposition, during its spreading) can be neglected^26,27^ and this is confirmed by our results (see Figure 7C). However, it is hardly to find the second stage of evaporation (at constant angle and decreasing contact area), excepting the system Cu0 (Figures 7A and 7C).

Therefore, we estimate the kinetics of only first stage of evaporation (constant contact length). For the diffusion controlled evaporation the time changes of droplet volume, d*V*/d*t*, can be expressed as^27^
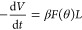
4where *L* is the contact radius, β and *F(θ)* are defined as
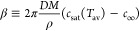
5
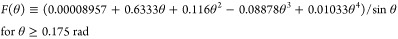
6In our case θ is always above 0.175 rad, therefore we do not show *F(θ)* for θ < 0.175 rad.

In eq 5, *D*, *M* and ρ are the diffusion coefficient of H_2_O vapor in air, water molar mass, and liquid density, respectively, *c*_sat_ is the molar concentration of saturated vapor at the temperature *T*_av_ which is the average temperature of a surface. Finally, *c*_*∞*_ = *Hc*_sat_(*T*_∞_) is the molar concentration of vapor, *T*_∞_ and *H* are the temperature and humidity of air far from the surface, respectively.

As the droplet volume can be expressed as a function of *L* and θ:^27^
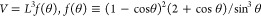
7eq 4 for *L* = const. can be expressed as
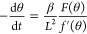
8To find β and θ_0_ (θ at *t* = 0) which give the smallest deviation of θ(*t*) obtained by solving eq 8 calculated from the experimental θ, we applied the Nelder–Mead method. The initial period where there are changes of *L* are excluded from the fitting. Thus, θ_0_ is close to the advancing contact angle. The results are presented in SI Table S1 and, for selected systems, on Figure 7C. It can be seen that the data fit is reasonably good as indicate the values of the determination coefficient (*R*^*2*^ in the range 0.995–0.9999).

The time dependence of approximated contact angles is slightly more steep than the experimental one. It does not seem to be caused by the decrease in the driving force of evaporation, *X* ≡ *c*_sat_(*T*_av_) – *c*_∞_, which in the calculations is assumed to be constant. As the droplet decreases *T*_av_ should not decrease because the distance between the metal plate and the droplet surface decreases and the heat transport to the surface should be faster. The value of *c*_∞_ should be practically constant because the volume of evaporated liquid is very small compared to the compartment where the droplet is deposited. The parameter β practically does not depend on the kind of surface on which the droplet was deposited; there is no correlation between β and θ_0_ or *L* and the standard deviation does not exceed 5% of the average value of β. Thus, it confirms that β depends only on the driving force of evaporation, *X* ≡ *c*_sat_(*T*_av_) – *c*_∞_, and the parameters related to vapor (*D*) and liquid (*M*, *ρ*). In SI Table S1, *X* divided by the saturated vapor concentration at the measurement temperature, *c*_sat_(*T*_m_) is also shown. The value *c*_sat_(*T*_m_) can be treated as the maximum force of evaporation, thus *X*/*c*_sat_(*T*_m_) represents the fraction of that force. Because *T*_av_ < *T*_m_ and *c*_∞_ > 0 *X* is below 50% of the maximum force.

There are correlations observed between θ_0_ and *L* (the higher is *θ*_*0*_, the lower *L* is). They are obvious considering the fact that the initial volumes of droplets were almost the same. Summing up, the results of droplet evaporation on studied Cu surfaces are described by the model proposed by Semenov et al.^26^ very well. Generally, adsorbed hydrocarbons influence the WCA, and prolong the process but do not change the mechanism of droplet evaporation from Cu surface.

### 3.3 Droplet Freezing

**Figure 8:**
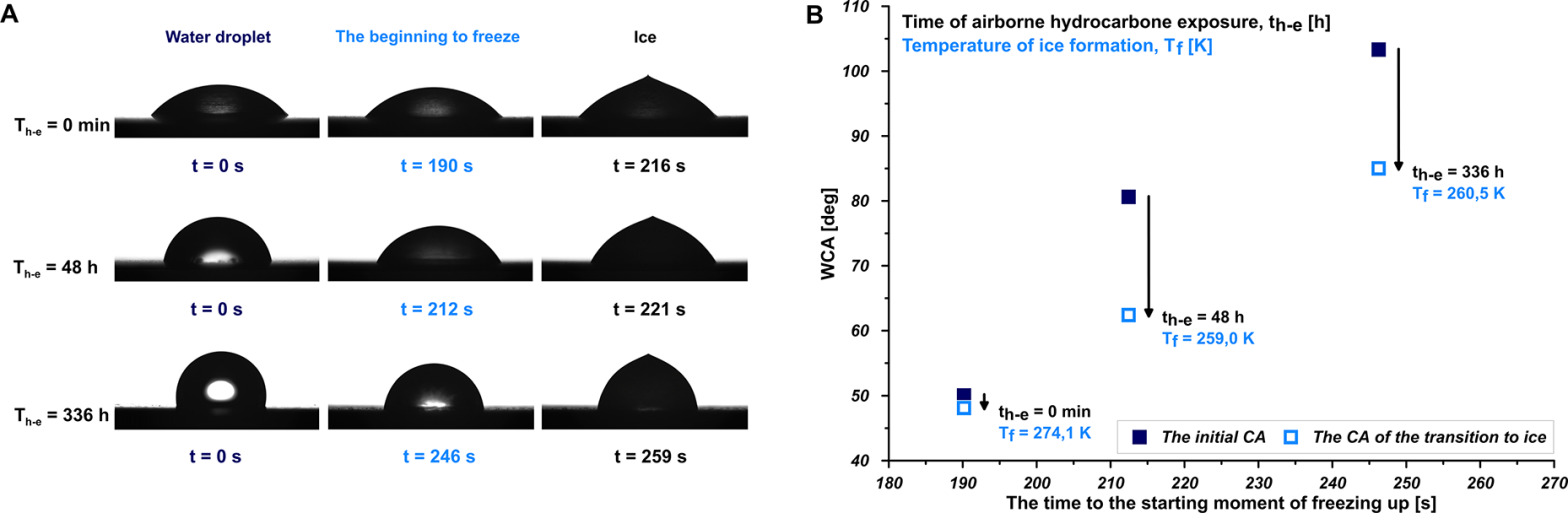
Behavior of water droplets for different times of exposition of *Cu-2000-Air* sample for airborne hydrocarbons; the pictures were taken at the beginning of the surface cooling process, at the start of freezing up, i.e. the moment of the start of ice nucleation from the bottom of the droplet to its top, and at the point of ice formation in the whole volume of the droplet (A), together with the change of WCA with the time of cooling down the surface from the ambient temperature (25 °C) to the moment of freezing up (B).

The droplet freezing experiment is very informative from the material and application points of view. It is clear that the presence or lack of the adsorbed airborne hydrocarbons have a significant influence on the surface resistance to cooling (Figure 8) and ice formation. For the material that has no contact with the airborne hydrocarbons (time for hydrocarbons exposition, *t*_*h-e*_ = 0 min) the lowest contact angle (WCA = 50.1°) as well as the shortest time (*t* = 190 s, start of freezing up, full ice formation after 216 s) required to freeze the materials was observed (Figure 8A). Then after 2 days of exposure (*t*_*h-e*_ = 48 h) of the sample on the air 61% of WCA increase was seen (Figure 8A). However, a substantial difference was observed after 2 weeks of contacting air atmosphere (*t*_*h-e*_ = 336 h). Due to the formation of a layer of airborne hydrocarbons, the value of the WCA exceeded the hydrophobicity level and was equal to 103.3°. Moreover, in this case the time required for freezing of a droplet on the surface was also prolonged to 259 s (Figure 8A). The results collected in Figure 8B show, that the temperature of the start of ice formation decreases with the time to surface exposure for the atmosphere. It means that the hydrocarbons layer acts as a protective layer due to its own heat capacity.

At the next stage, we used a theoretical model to check the evolution of profiles of frozen droplets. As the Bond number for our droplets is within the range 0.28–0.45 the gravity effect on the droplet shape can be neglected. Although a more advanced description of the freezing of a droplet exists,^28^ for the fitting of the frozen sessile droplet, we decided to apply a simple model for zero gravity and dynamic growth angle.^29,30^ In this approach mass conservation demands that the rate of change of liquid volume is related to the solidification rate (d*H*/d*t*) via:
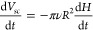
9where *H(t)* is the height of the solid–liquid interface, *V*_SC_ is the volume of a spherical cap, ν = *ρ*_s_/*ρ*_l_, *ρ*_s_, *ρ*_l_ are the densities of solid (ice), and liquid water, respectively. The radial coordinate of the trijunction line evolves as
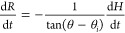
10where θ is the apparent contact angle. The dynamic growth angle *θ*_*i*_ is related to the slip velocity *V*_*S*_ via^29^
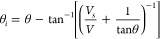
11

**Figure 9:**
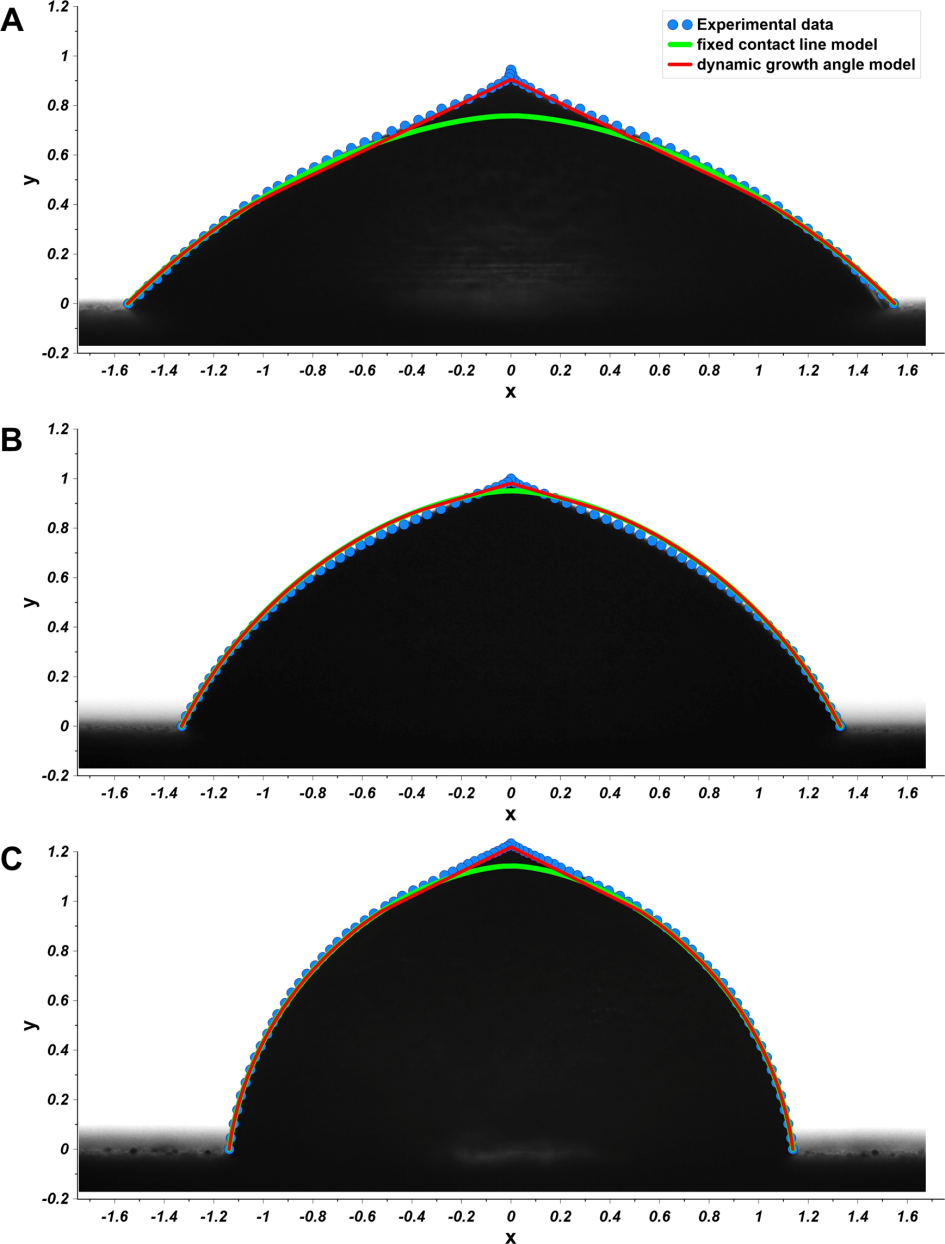
Droplet profiles (blue points) fitted by dynamic growth angle model and fixed contact line model, for the systems *Cu-2000-Air-0 min* (A), *Cu-2000-Air-48 h* (B), and *Cu-2000-Air-336 h* (C). The droplets heights and widths were normalized taking into account the droplet B.

In the above *V* = d*H*/d*t*, and we use
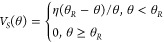
12where *θ*_*R*_ and η are fitting parameters. By setting *θ*_*i*_= 0 one recovers the fixed contact line model. While the latter model is purely geometrical, that is, the final shape is independent of the solidification rate, the former, dynamic growth angle model requires specification of the rate of solidification. Here we assume that
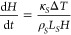
13where *κ*_*S*_ is the thermal conductivity, *L*_*S*_ is the latent heat of solidification, *ρ*_*S*_ is the density of ice, and *ΔT* is the difference between the freezing temperature and the temperature of the cold plate.^29^Equations 9–13 together with a relation for *V*_*SC*_ form a set of differential-algebraic equations that has to be solved numerically. Numerical details of the fits are collected in SI Table S2 and the results are presented in Figure 9. We note that the incorporation of nonzero growth angle significantly improves the quality of the fits. Clearly, eq 13 represents a rather crude approximation to the actual experimental conditions. However, the good quality of the obtained fits suggests that the final shapes of the droplets are insensitive to the precise details of the solidification front description, at least for the cooling rates used in our experiment (SI Figure S3).^31,32^

## Conclusions

The application of modern Fringe Projection Phase-Shifting method and a new cell allowing the in situ measurement of WCA in a controlled atmosphere after a sample desorption, leads to the conclusion that Cu (1 1 1) surface is hydrophilic with WCA = 34°. This is in accordance with the shapes of water adsorption isotherms reported in the literature. When subjected to atmosphere WCA increases due to adsorption of the airborne hydrocarbons. The application of MD simulations provides the WCA values equal to 52° (for (1 1 0)) and 44° (for Cu (0 0 1)), thus WCA strongly depends on the type of Cu planes. Airborne hydrocarbons strongly influence droplet evaporation and freezing, prolonging the evaporation and decreasing ice-formation temperature. The evaporation process is well described by the model of Semenov,^26^ whereas the final shapes of frozen sessile droplets are well characterized using the dynamic growth angle model.^29^

## Supporting Information Available

XRD spectra of studied (1 1 1) copper. Fringe projection phase-shifting method. MD simulation details. Water evaporation fitting results (PDF)

## References

[ref1] DROPLET. Droplet Wetting and Evaporation: From Pure to Complex Fluids; Brutin D., Ed.; Elsevier/AP, Academic Press: Amsterdam Boston Heidelberg, 2015.

[ref2] Zhang C.; Zhou W.; Wang Q.; Wang H.; Tang Y.; Hui K. S. Comparison of Static Contact Angle of Various Metal Foams and Porous Copper Fiber Sintered Sheet. Appl. Surf. Sci. 2013, 276, 377–382. 10.1016/j.apsusc.2013.03.101.

[ref3] Bizi-Bandoki P.; Valette S.; Audouard E.; Benayoun S. Time Dependency of the Hydrophilicity and Hydrophobicity of Metallic Alloys Subjected to Femtosecond Laser Irradiations. Appl. Surf. Sci. 2013, 273, 399–407. 10.1016/j.apsusc.2013.02.054.

[ref4] Vadgama B.; Harris D. K. Measurements of the Contact Angle between R134a and Both Aluminum and Copper Surfaces. Exp. Therm. Fluid Sci. 2007, 31 (8), 979–984. 10.1016/j.expthermflusci.2006.10.010.

[ref5] Li Z.; Wang Y.; Kozbial A.; Shenoy G.; Zhou F.; McGinley R.; Ireland P.; Morganstein B.; Kunkel A.; Surwade S. P.; Li L.; Liu H. Effect of Airborne Contaminants on the Wettability of Supported Graphene and Graphite. Nat. Mater. 2013, 12 (10), 925–931. 10.1038/nmat3709. 23872731

[ref6] Terzyk A. P.; Bryk P.; Korczeniewski E.; Kowalczyk P.; Zawadzka A.; Płóciennik P.; Wiśniewski M.; Wesołowski R. P. Water Nanodroplet on a Hydrocarbon “Carpet”—The Mechanism of Water Contact Angle Stabilization by Airborne Contaminations on Graphene, Au, and PTFE Surfaces. Langmuir 2019, 35 (2), 420–427. 10.1021/acs.langmuir.8b03790. 30562472

[ref7] Smith T. The Hydrophilic Nature of a Clean Gold Surface. J. Colloid Interface Sci. 1980, 75 (1), 51–55. 10.1016/0021-9797(80)90348-3.

[ref8] Bryk P.; Korczeniewski E.; Szymański G. S.; Kowalczyk P.; Terpiłowski K.; Terzyk A. P. What Is the Value of Water Contact Angle on Silicon?. Materials 2020, 13 (7), 1554 10.3390/ma13071554. PMC7177545 32230922

[ref9] Aria A. I.; Kidambi P. R.; Weatherup R. S.; Xiao L.; Williams J. A.; Hofmann S. Time Evolution of the Wettability of Supported Graphene under Ambient Air Exposure. J. Phys. Chem. C 2016, 120 (4), 2215–2224. 10.1021/acs.jpcc.5b10492. PMC4754094 26900413

[ref10] Yekta-Fard M.; Ponter A. B. Surface Treatment and Its Influence on Contact Angles of Water Drops Residing on Teflon and Copper. J. Adhes. 1985, 18 (3), 197–205. 10.1080/00218468508079683.

[ref11] Schrader M. E. Ultrahigh Vacuum Techniques in the Measurement of Contact Angles. I11. Water on Copper and Silver’. J. Phys. Chem. 1974, 78 (1), 87–89. 10.1021/j100594a017.

[ref12] Li J.; Zhou Y.; Wang W.; Xu C.; Ren L. Superhydrophobic Copper Surface Textured by Laser for Delayed Icing Phenomenon. Langmuir 2020, 36 (5), 1075–1082. 10.1021/acs.langmuir.9b02273. 31958954

[ref13] Nilsson M. A.; Daniello R. J.; Rothstein J. P. A Novel and Inexpensive Technique for Creating Superhydrophobic Surfaces Using Teflon and Sandpaper. J. Phys. D: Appl. Phys. 2010, 43 (4), 045301 10.1088/0022-3727/43/4/045301.

[ref14] Furmaniak S.; Gauden P. A.; Terzyk A. P.; Rychlicki G. Water Adsorption on Carbons — Critical Review of the Most Popular Analytical Approaches. Adv. Colloid Interface Sci. 2008, 137 (2), 82–143. 10.1016/j.cis.2007.08.001. 17919444

[ref15] Thommes M.; Kaneko K.; Neimark A. V.; Olivier J. P.; Rodriguez-Reinoso F.; Rouquerol J.; Sing K. S. W. Physisorption of Gases, with Special Reference to the Evaluation of Surface Area and Pore Size Distribution (IUPAC Technical Report). Pure Appl. Chem. 2015, 87 (9–10), 1051–1069. 10.1515/pac-2014-1117.

[ref16] Lee S.; Staehle R. W. Adsorption of Water on Copper, Nickel, and Iron. Corrosion 1997, 53 (1), 33–42. 10.5006/1.3280431.

[ref17] Sharma S. P.; Iii J. H. T. Adsorption of Water Vapor on Thin-gold Electroplate on Copper. J. Vac. Sci. Technol. 1977, 14 (3), 825–827. 10.1116/1.569277.

[ref18] Platzman I.; Saguy C.; Brener R.; Tannenbaum R.; Haick H. Formation of Ultrasmooth and Highly Stable Copper Surfaces through Annealing and Self-Assembly of Organic Monolayers. Langmuir 2010, 26 (1), 191–201. 10.1021/la902006v. 19715329

[ref19] White M. L.; Drobek J. The Effect of Residual Abrasives on the Wettability of Polished Gold Surfaces. J. Phys. Chem. 1966, 70 (11), 3432–3436. 10.1021/j100883a010.

[ref20] Abascal J. L. F.; Vega C. A General Purpose Model for the Condensed Phases of Water: TIP4P/2005. J. Chem. Phys. 2005, 123 (23), 234505 10.1063/1.2121687. 16392929

[ref21] Jorgensen W. L.; Maxwell D. S.; Tirado-Rives J. Development and Testing of the OPLS All-Atom Force Field on Conformational Energetics and Properties of Organic Liquids. J. Am. Chem. Soc. 1996, 118 (45), 11225–11236. 10.1021/ja9621760.

[ref22] Friedrichs M. S.; Eastman P.; Vaidyanathan V.; Houston M.; Legrand S.; Beberg A. L.; Ensign D. L.; Bruns C. M.; Pande V. S. Accelerating Molecular Dynamic Simulation on Graphics Processing Units. J. Comput. Chem. 2009, 30 (6), 864–872. 10.1002/jcc.21209. 19191337 PMC2724265

[ref23] Eastman P.; Pande V. S. Constant Constraint Matrix Approximation: A Robust, Parallelizable Constraint Method for Molecular Simulations. J. Chem. Theory Comput. 2010, 6 (2), 434–437. 10.1021/ct900463w. 20563234 PMC2885791

[ref24] Eastman P.; Friedrichs M. S.; Chodera J. D.; Radmer R. J.; Bruns C. M.; Ku J. P.; Beauchamp K. A.; Lane T. J.; Wang L.-P.; Shukla D.; Tye T.; Houston M.; Stich T.; Klein C.; Shirts M. R.; Pande V. S. OpenMM 4: A Reusable, Extensible, Hardware Independent Library for High Performance Molecular Simulation. J. Chem. Theory Comput. 2013, 9, 9 10.1021/ct300857j. PMC3539733 23316124

[ref25] Humphrey W.; Dalke A.; Schulten K. VMD: Visual Molecular Dynamics. J. Mol. Graphics 1996, 14 (1), 33–38. 10.1016/0263-7855(96)00018-5. 8744570

[ref26] Semenov S. Evaporation of Sessile Water Droplets: Universal Behaviour in Presence of Contact Angle Hysteresis. Colloids Surf., A 2011, 391 (1–3), 135–144. 10.1016/j.colsurfa.2011.07.013.

[ref27] Perrin L.; Pajor-Swierzy A.; Magdassi S.; Kamyshny A.; Ortega F.; Rubio R. G. Evaporation of Nanosuspensions on Substrates with Different Hydrophobicity. ACS Appl. Mater. Interfaces 2018, 10 (3), 3082–3093. 10.1021/acsami.7b15743. 29268600

[ref28] Picknett R. G.; Bexon R. The Evaporation of Sessile or Pendant Drops in Still Air. J. Colloid Interface Sci. 1977, 61 (2), 336–350. 10.1016/0021-9797(77)90396-4.

[ref29] Zhang C.; Zhang H.; Fang W.; Zhao Y.; Yang C. Axisymmetric Lattice Boltzmann Model for Simulating the Freezing Process of a Sessile Water Droplet with Volume Change. Phys. Rev. E: Stat. Phys., Plasmas, Fluids, Relat. Interdiscip. Top. 2020, 101 (2), 023314 10.1103/PhysRevE.101.023314. 32168660

[ref30] Anderson D. M.; Worster M. G.; Davis S. H. The Case for a Dynamic Contact Angle in Containerless Solidification. J. Cryst. Growth 1996, 163 (3), 329–338. 10.1016/0022-0248(95)00970-1.

[ref31] Snoeijer J. H.; Brunet P. Pointy Ice-Drops: How Water Freezes into a Singular Shape. Am. J. Phys. 2012, 80 (9), 764–771. 10.1119/1.4726201.

[ref32] Zhang X.; Wu X.; Min J.; Liu X. Modelling of Sessile Water Droplet Shape Evolution during Freezing with Consideration of Supercooling Effect. Appl. Therm. Eng. 2017, 125, 644–651. 10.1016/j.applthermaleng.2017.07.017.

