# Supplementary material for: Revisiting Wetting, Freezing, and Evaporation Mechanisms of Water on Copper
Source: ACS Appl Mater Interfaces. 2021 Jul 28;13(31):37893–903. doi: 10.1021/acsami.1c09733 (PMC8397239; doi:10.1021/acsami.1c09733)
Supplement: Supplementary file 1 — am1c09733_si_001.pdf [file am1c09733_si_001.pdf]

Supporting information

Revisiting Wetting, Freezing and Evaporation Mechanisms of Water on Copper

Emil Korczeniewski[1], Paweł Bryk[2], Stanisław Koter[3], Piotr Kowalczyk[4], Wojciech Kujawski[3], Joanna Kujawa[3,] and Artur P. Terzyk[1,*]*

1) Faculty of Chemistry, Physicochemistry of Carbon Materials Research Group, Nicolaus Copernicus University in Toruń, Gagarin Street 7, 87-100 Toruń, Poland,

2) Faculty of Chemistry, Chair of Theoretical Chemistry, Maria Curie - Skłodowska University, 20 - 031 Lublin, Poland,

3) Faculty of Chemistry, Department of Physical Chemistry and Physical Chemistry of Polymers, Nicolaus Copernicus University in Toruń, Gagarin Street 7, 87-100 Toruń, Poland,

4) College of Science, Health, Engineering and Education, Murdoch University, WA 6150, Australia.

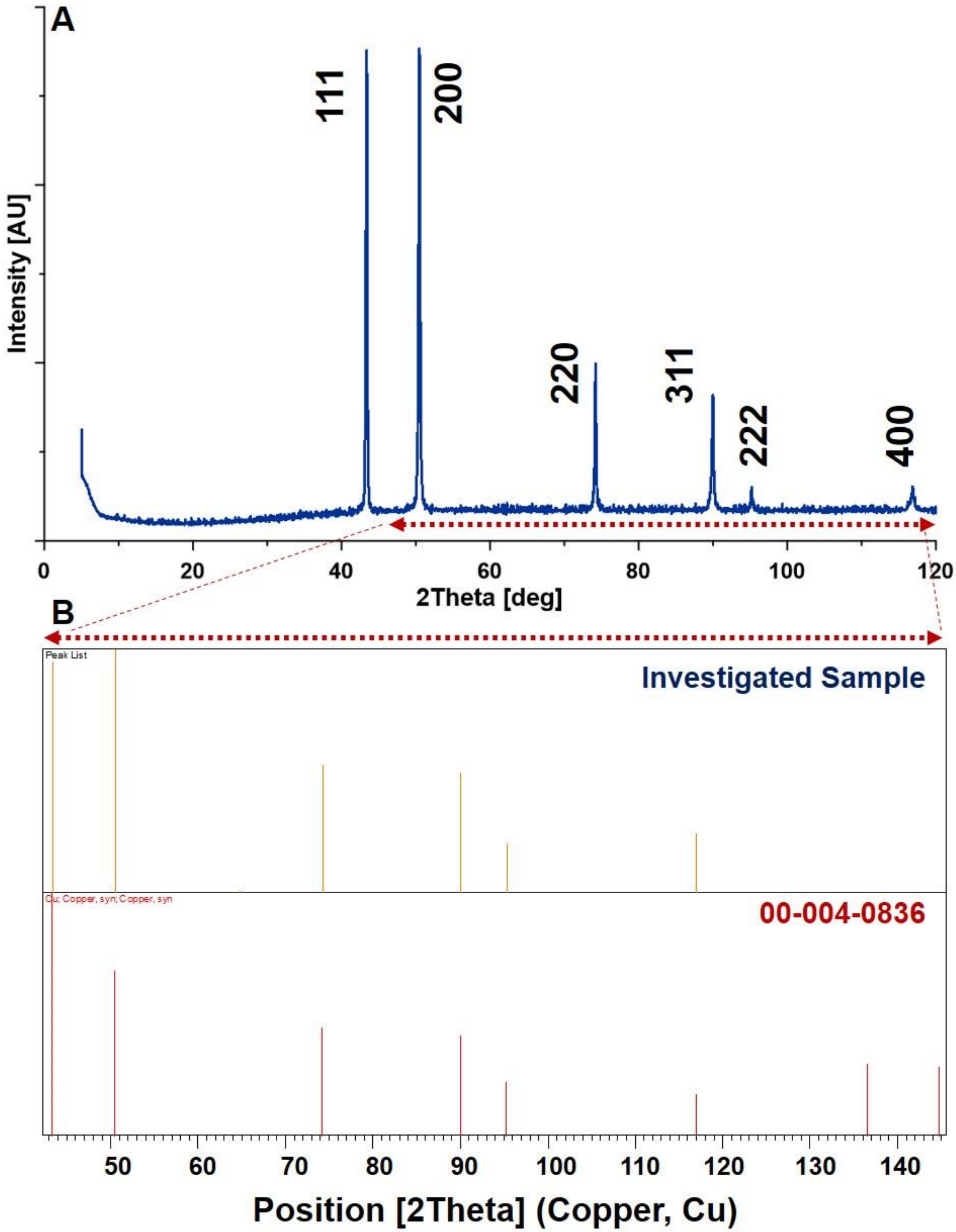

Figure S1. The XRD spectra of studied copper with the peaks position on the pattern (00-0040-0836).

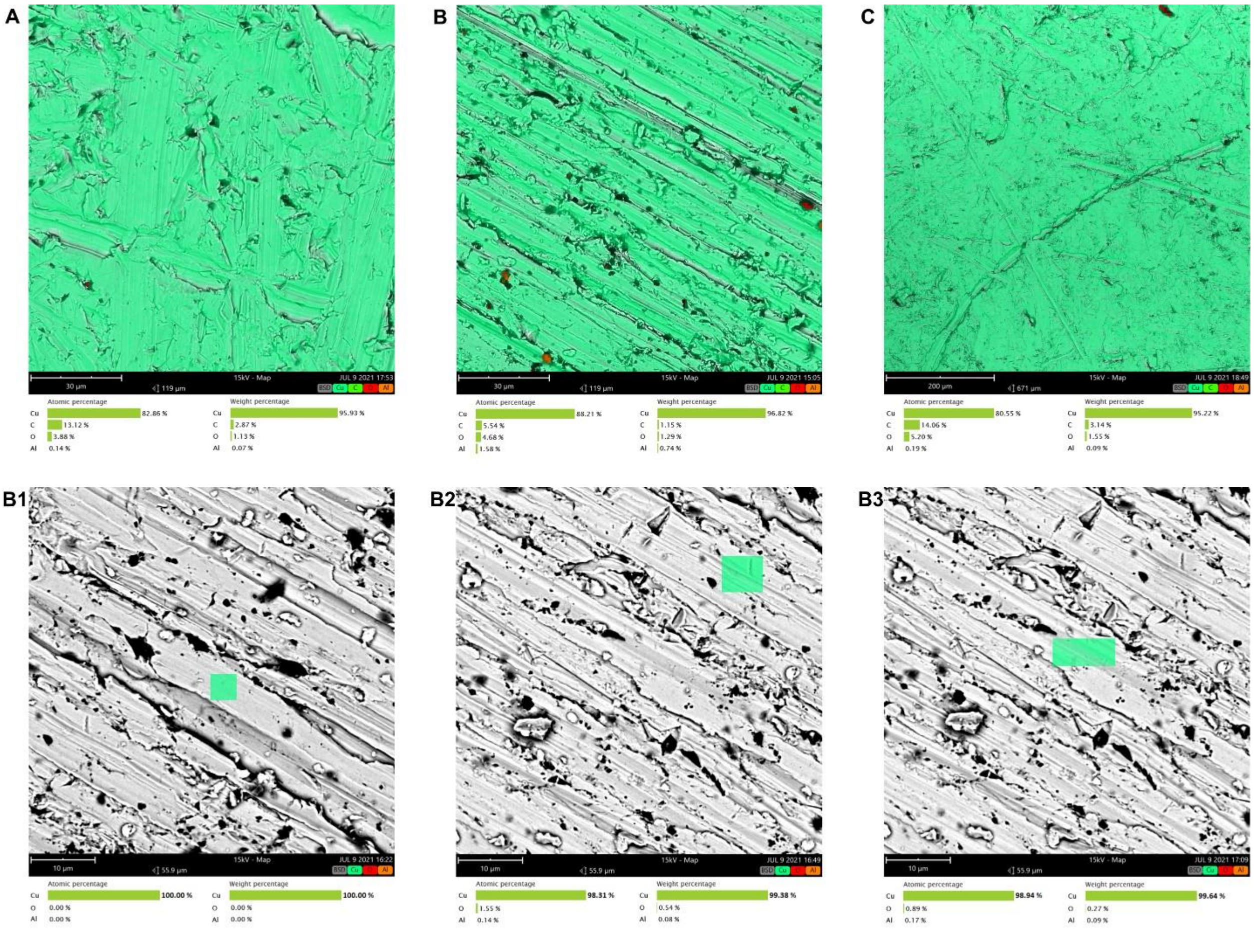

Figure S2. EDS analysis of the initial copper sample (Cu-0-Air) (A, C) and surface freshly exposed by treatment with sandpaper (Cu-2000-Air-0min) (B).

Figures A and C show the elemental EDS analysis for the initial copper sample (Cu-0-Air). EDS analysis shows that on the surface cleaned with an argon stream there are only traces of corundum, but only in not very numerous fragments of hard-to-penetrate deep grooves, in which the Al_2O_3 grains were jammed (B). The outer surface of the copper samples shows zero (B1), or within the measurement error, of traces of the element aluminum (compare B2-3 with A and C). EDS analysis also shows a remarkably large amount of the hydrocarbon-derived element C on the sample saturated with Cu-0-Air (A and C) compared to a much smaller amount of this element on the surface freshly exposed by treatment with sandpaper (Cu-2000-Air-0min) (B).

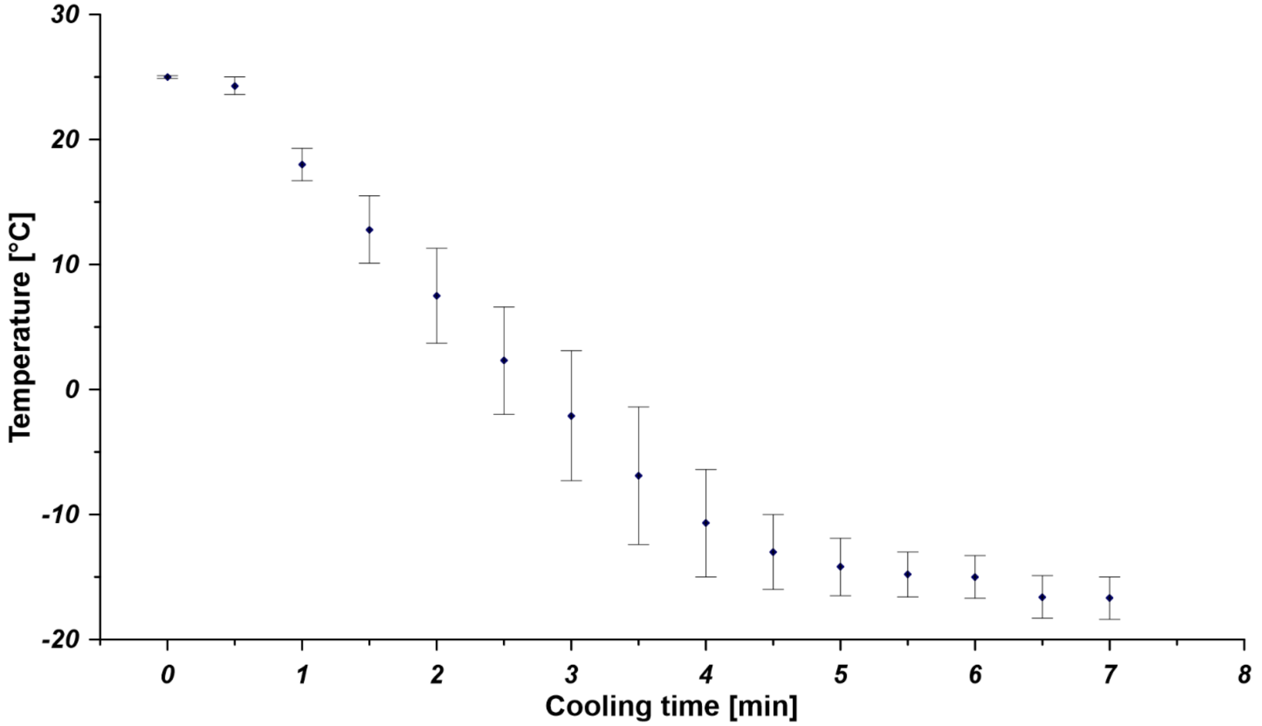

Figure S3. The relation between temperature and the time of cooling.

Fringe projection phase-shifting method

The contact angle corrected by roughness it is the value of the contact angle on the Young surface. An implementation of the fringe projection phase-shifting method is giving a unique opportunity to analyze surface roughness and contact angle of the same location of the sample. As a result, the contact angle on the rough surface, flat surface, roughness, and area factor of roughness are determined.

In Fig. S4 and Fig. S5 the principal of the method is presented.

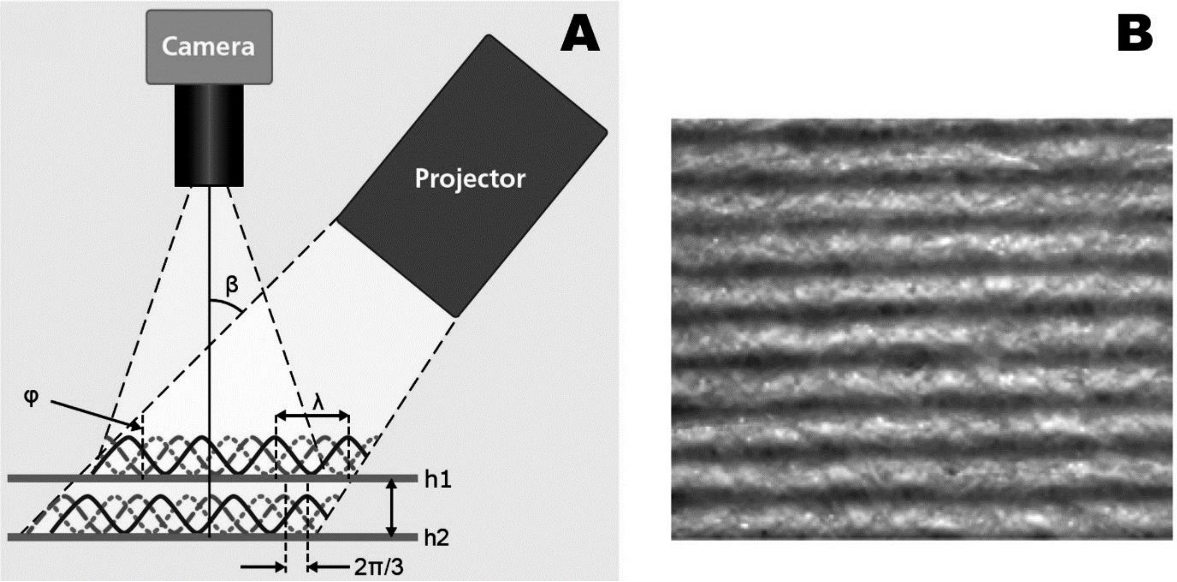

Figure S4. Fringe Projection Phase-Shifting schematics (A) and an example of a surface with the projected pattern (B). A sinusoidal pattern is sequentially projected on the sample surface and a camera is utilized to capture the fringe patterns and reconstruct the 3D image by phase-shift coding.

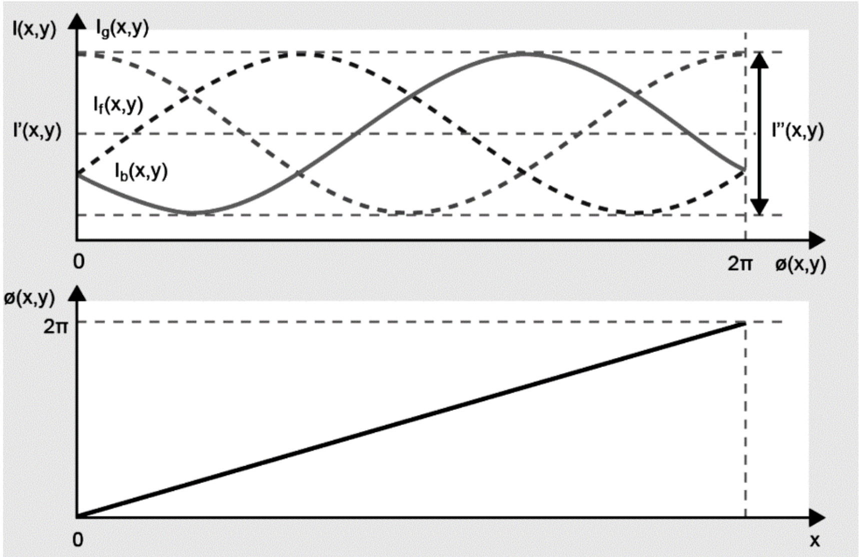

Figure S5. In the sinusoidal phase-shifting method a series of phase-shifted sinusoidal patterns are recorded (top), from which the phase information at every pixel is obtained (bottom). The phase shift correlates with sample surface topography from pixel to the pixel that defines the resolution.

As illustrated in Fig. S4 the 3D topography module consists of a projector with a LED light source and a slide with sinusoidal fringe patterns on a grey-scale. These illumination patterns are sequentially projected onto the studied surface, and a digital camera captures the fringe patterns from which the 3D shape of the object is reconstructed by phase-shift coding. This enables pixel level measurement resolution. In the 3D Topography Module, the pixel size is 1.1 μm x 1.1 μm for analysis of micron-scale surface features.

The sinusoidal fringes can be expressed by:

$$I_n(x,y) = a + b\cos\left(\frac{2px}{p} + \varphi_0 + \delta_n\right)$$

where (x, y) is the coordinate in the slide frame plane, a is background intensity, b is amplitude modulation, p is the sinusoidal grating wavelength, φ_0 is the additional phase shift caused by the surface height and δ_n is the phase shift from the slide movement.

As an example, for a color pattern with red, green, and blue light, it can be demonstrated the case of three divided wavelengths where I_r, I_g, and I_b are the corresponding intensities for each of the

colors. The phase shifts can be plotted as in Fig. S3. Then the spatial phase shift can be expressed with the following equation:

$$\varphi(x,y) = \arctan\left|\sqrt{\frac{I_r - I_b}{2I_g - I_r - I_b}}\right|$$

The phase shift indicates the horizontal coordinate, i.e. the height differences in every pixel providing the sample topography.

MD simulation details

We model the copper surface (ca. 36 000 atoms) as a collection of atoms arranged on the fcc lattice with constant of 3.6147 Å. Cu atoms are assumed to be motionless and interact with water and alkane via the Lennard-Jones (12-6) potential. The Cu diameter σ_{Cu} = 2.56 Å, while the energy parameter ε_{Cu-Cu} is determined empirically by matching the experimental water contact angle. We investigate two copper surfaces, namely (0 0 1) and (1 1 1). The systems with (0 0 1) Cu plane consist of either 36432 atoms arranged in a slab of dimensions 498.8286 Å × 43.3764 Å × 18.0735 Å, and used in modeling cylindrical drops on copper surfaces, or 79200 atoms, arranged in a slab of dimensions 216.882 Å × 216.882 Å ×18.0735 Å, and used in modeling spherical drops on copper surfaces. Likewise, the systems with (1 1 1) Cu plane comprise of either 38940 atoms arranged in a slab of dimensions 498.415905 Å × 44.27085 Å × 16.695584 Å, or 83006 atoms arranged in a slab of dimensions 217.258215 Å × 216.927165 Å × 16.695584 Å.

We assumed the additivity of diameters and the Lorentz-Berthelot mixing rule. The Coulomb and LJ interactions between atoms separated by three bonds within the same molecule were scaled down by multiplying them by 0.833333 and 0.5, respectively. All dispersion interaction has a cutoff radius r_{cut} = 1.5nm and the same distance was used to switch from real-space to Fourier space calculations of the electrostatics.

The computational procedure is analogous to that used in our previous paper.[6] The solid fluid interaction parameter was determined by matching the WCA of a cylindrical drop to the experimental value. To this end 3900 TIP4P/2005 water molecules were placed on top of the

copper surface. After preemptive energy minimization the drop was equilibrated for 10 ns, and the averages were gathered for up to 40 ns.

Water evaporation fitting results

Table S1. The results of eq 8 fitting to experimental data.

System	T_m [°C]	L [mm]	time range of fit [s]	R^2	θ_0 [rad]	β [mm^2/s]	$c_{sat}(T_m)$ [mol/m^3]	$X/c_{sat}(T_m)$ a)
Cu-0-Air-infinity-1st	20.5	1.19	0-1600	0.9991	1.976	0.00298	0.988	0.441
Cu-0-Air-infinity-2nd	20.5	1.18	0-1000	0.9990	1.980	0.00293	0.988	0.434
Cu-0-Air-infinity-3rd	20.5	1.18	0-1000	0.9995	1.989	0.00316	0.988	0.468
Cu-2000-Air-0min-1st	21.5	1.55	0-1000	0.9999	1.326	0.00306	1.047	0.425
Cu-2000-Air-0min-2nd	21.5	1.71	0-600	0.9990	0.918	0.00311	1.047	0.431
Cu-2000-Air-20min-1st	20.5	1.40	0-1000	0.9981	1.624	0.00299	0.988	0.444
Cu-2000-Air-20min-2nd	20.5	1.50	0-1000	0.9998	1.326	0.00283	0.988	0.420
Cu-2000-Air-20min-3rd	20.5	1.53	0-800	0.9998	1.323	0.00283	0.988	0.420
Cu-2000-Air-20min-4th	20.5	1.54	0-800	0.9995	1.442	0.00309	0.988	0.458
Cu-2000-Air-20min-5th	20.5	1.43	40-800	0.9981	1.446	0.00311	0.988	0.461
Cu-2000-Air-8mths	21.5	1.24	160-1600	0.9986	1.910	0.00309	1.047	0.428
Cu-2000-Vac-Air-8mths	20.5	1.08	1500	0.9971	2.116	0.00314	0.988	0.466

Cu-2000-Air-93hrs-1st	21	1.32	110-1600	0.9982	1.764	0.00277	1.018	0.397
Cu-2000-Air-93hrs-2nd	21	1.07	0-1600	0.9976	2.113	0.00307	1.018	0.440
Cu-2000-Air-93hrs-3th	21	1.50	40-1200	0.9981	1.556	0.00333	1.018	0.477
Cu-2000-Air-93hrs-4th	21.5	1.27	0-1400	0.9984	1.841	0.00311	1.047	0.431
Cu-2000-Air-93hrs-5th	21.5	1.18	160-1600	0.9954	1.979	0.00311	1.047	0.431
					β_{av} =	0.00304	$(X/c_{sat,m})_{av}$ =	0.440
					std. dev. =	0.00014	std. dev. =	0.021

[a] $X \equiv c_{sat}(T_{av}) - c_\infty$ was calculated from eq 5; c_{sat} was calculated from $c_{sat} = p_{sat}/RT$, p_{sat} was taken from Engineering ToolBox, (2004). Water - Saturation Pressure. [online] Available at: https://www.engineeringtoolbox.com/water-vapor-saturation-pressure-d_599.html [7th April 2021].The temperature dependence of liquid water density was taken from Söhnel O. and Novotný P., Densities of Aqueous Solutions of Inorganic Substances, Academia Prague, 1985.

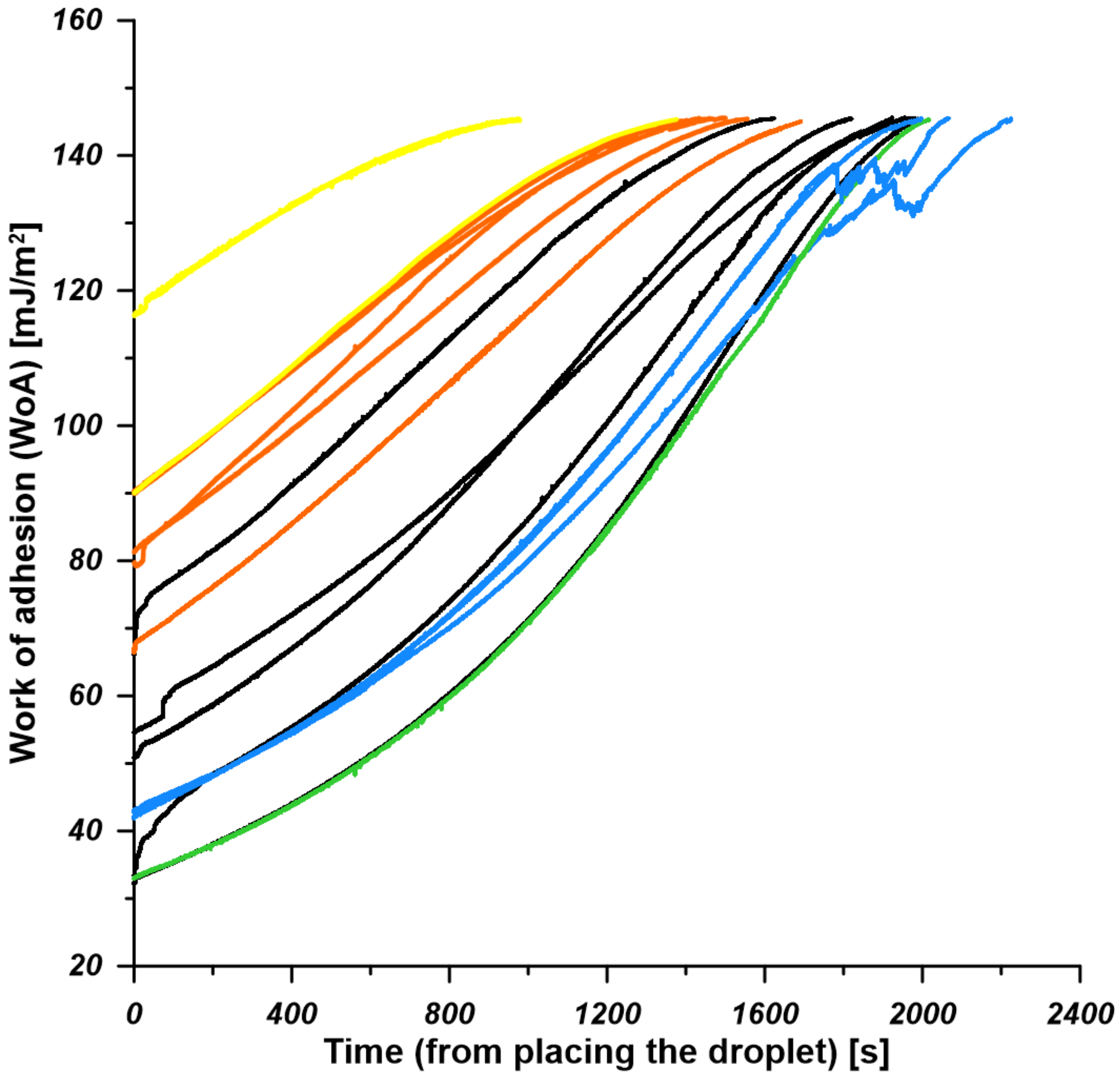

Figure S6. The changes in the values of the work of adhesion, corresponding to the evaporation curves shown in Fig.6.

Table S2. The parameters of the fit of the models applied for droplet freezing.

<table>
<tr><td>System</td><td>R_0 [mm]</td><td>θ_0 [rad]</td><td rowspan="4">κ_S: 2.26 [W $m^{-1}K^{-1}$]
L_S: 333.4 [kJ kg^{-1}]
ρ_S: 918.2 [kg m^{-3}]
ΔT: 10 [K]
η: 1.1 [mm s^{-1}]
θ_R: 0.186π [rad]
ν: 0.917</td></tr>
<tr><td>*Cu-2000-Air-0min*</td><td>2.06</td><td>0.787</td></tr>
<tr><td>*Cu-2000-Air-48hrs*</td><td>1.77</td><td>1.100</td></tr>
<tr><td>*Cu-2000-Air-336hrs*</td><td>1.52</td><td>1.437</td></tr>
</table>
